# Biological Properties of Oleanolic Acid Derivatives Bearing Functionalized Side Chains at C-3

**DOI:** 10.3390/ijms25158480

**Published:** 2024-08-03

**Authors:** Gianfranco Fontana, Natale Badalamenti, Maurizio Bruno, Filippo Maggi, Federica Dell’Annunziata, Nicoletta Capuano, Mario Varcamonti, Anna Zanfardino

**Affiliations:** 1 Department of Biological, Chemical and Pharmaceutical Sciences and Technologies (STEBICEF), University of Palermo, Viale delle Scienze, 90128 Palermo, Italy; gianfranco.fontana@unipa.it (G.F.); natale.badalamenti@unipa.it (N.B.); maurizio.bruno@unipa.it (M.B.); 2 NBFC, National Biodiversity Future Center, 90133 Palermo, Italy; 3 Centro Interdipartimentale di Ricerca “Riutilizzo Bio-Based degli Scarti da Matrici Agroalimentari” (RIVIVE), University of Palermo, Viale delle Scienze, 90128 Palermo, Italy; 4 Chemistry Interdisciplinary Project (ChIP) Research Center, University of Camerino, Via Madonna delle Carceri, 62032 Camerino, Italy; 5 Department of Medicine, Surgery and Dentistry, “Scuola Medica Salernitana”, University of Salerno, 84081 Baronissi, Italy; federica.dellannunziata@unicampania.it (F.D.); niccapuano@unisa.it (N.C.); 6 Department of Experimental Medicine, University of Campania Luigi Vanvitelli, 80138 Naples, Italy; 7 Department of Biology, University of Naples, Federico II, Via Cinthia, 80126 Naples, Italy; varcamon@unina.it (M.V.); anna.zanfardino@unina.it (A.Z.)

**Keywords:** triterpene derivatives, oleanolic acid, antibacterial activity, SARS-CoV-2, PV-1

## Abstract

Triterpene acids are a class of pentacyclic natural carboxylic compounds endowed with a variety of biological activities including antitumor, antimicrobial, and hepatoprotective effects. In this work, several oleanolic acid derivatives were synthesized by structurally modifying them on the C-3 position. All synthesized derivatives were evaluated for possible antibacterial and antiviral activity, and among all the epimers, **6** and **7** demonstrated the best biological activities. Zone-of-inhibition analyses were conducted against two strains, *E. coli* as a Gram-negative and *S. aureus* as a Gram-positive model. Subsequently, experiments were performed using the microdilution method to determine the minimum inhibitory concentration (MIC). The results showed that only the derivative with reduced hydrogen bonding ability on ring A possesses remarkable activity toward *E. coli*. The conversion from acid to methyl ester implies a loss of activity, probably due to a reduced affinity with the bacterial membrane. Before the antiviral activity, the cytotoxicity of triterpenes was evaluated through a 3-(4,5-dimethylthiazol-2-yl)-2,5-diphenyltetrazolium bromide (MTT) assay. Samples **6** and **7** showed less than 50% cytotoxicity at 0.625 and 1 mg/mL, respectively. The antiviral activity against SARS-CoV-2 and PV-1 did not indicate that triterpene acids had any inhibitory capacity in the sub-toxic concentration range.

## 1. Introduction

Triterpene acids are a class of pentacyclic natural carboxylic acids endowed with a variety of biological activities including antitumor, antimicrobial, and hepatoprotective effects. They possess a basic set of three functional groups that are the carboxylic moiety at C-28, an alkene moiety at C-11,12, and a *β*-hydroxyl fragment at C-3. Several other functional groups, including hydroxyl and carboxyl, can decorate other positions of the pentacyclic carbon framework [1]. Even today, despite the important results obtained through drugs of synthetic origin, naturally sourced products are fundamental for the development and discovery of new pharmacological agents. In fact, approximately 80% of existing anticancer agents are inspired by or come from metabolism of plants, algae, or sponges [2]. Interest in triterpenoids has significantly increased in the last twenty years, precisely due to their chemical complexity, the presence of different functionalities, and their biological versatility [3]. Many triterpenes such as bryonolic acid, butyrospermol, lupeol, 1*β*-hydroxyaleuritolic acid 3-*p*-hydroxybenzoate, and isotirucallol have proven to be excellent antiproliferative [4,5,6], antineoplastic [7,8], and antiviral agents [9,10].

In this contest, we have been working for a couple of years on the antitumor activity of semisynthetic derivatives of oleanolic acid (**1**). It has been demonstrated that the introduction of a side chain at C-3 of the natural compound can deeply modify the biological efficacy of the molecule by a complex pattern of effects that include solubility, biological membrane permeability, and interaction with specific target proteins [1]. For example, an inverse relationship was observed between the hydrophilicity of a three-carbon side chain introduced in place of the H-3 of **1** and the cytotoxic effect of the molecule on HL60 cells, both resistant and sensitive [1]. The compound with a 2′,3′-dihydroxypropyl- moiety at C-3 was less active than the one bearing an allyl moiety. Furthermore, conjugation with appropriate peptides, and, in particular, the p10 peptide, has also been shown to improve the bioavailability, permeability, and pharmacological efficacy of oleanolic acid [11].

The antibacterial activity of triterpene acids has been widely investigated by several authors. This activity is exerted through a rather complex mechanism of action that includes influences on the gene expression and regulation of the peptide–glycans turnover, biofilm formation, and cell autolysis [12]. Other studies have shown that triterpene acids exert their antibacterial activity through the disruption of the bacterial cell membrane. Rebamang et al. highlighted how some terpenes isolated from the stem bark of *Protorhus longifolia* (Benrh.) Engl. affect the integrity of microbial cell membrane [13]. Additionally, some studies suggest that triterpene acids may have a synergistic effect when combined with traditional antibiotics or with each other, enhancing the overall efficacy of the treatment. For example, Wang et al. demonstrated that ursolic acid owns a synergistic effect with ampicillin and tetracycline against *B. cereus* and *S. aureus* bacterial strains [14]. 

It has been highlighted that the presence of hydroxyl functionalities at different positions of the A ring of triterpene oleane and ursolic acid derivatives can sensibly improve the antibacterial activity of these derivatives toward Gram-positive bacteria [15]. Other modifications at the C-3 of ring A were investigated to improve the antibacterial activity; these include the introduction of nitrogen-containing heterocyclic side chains [16] as well as nitrogen-containing side chains at C-28 [17,18]. Moreover, several studies have shown that some triterpenoids (oleanane, ursane, lupane, dammarane, lanostane, and cycloartane) recorded antiviral potential, with the high performance associated with tetracyclic and pentacyclic triterpenoids [19,20,21]. Sasaki et al. showed that glycyrrhizin acid, a pentacyclic triterpenoid isolated from licorice root, effectively inhibited HIV replication by increasing the number of OKT4 lymphocytes and reducing protein kinase C activity [22].

Considering these data, it was decided to study the antibacterial potential of a series of synthetic oleanolic acid derivatives, with modifications that focused on the C-3 position, against two strains of *E. coli* as a Gram-negative and *S. aureus* as a Gram-positive model by evaluating the minimum inhibitory concentration (MIC) for bacterial growth, and the possible antiviral activity against SARS-CoV2 and PV-1. Part of the molecules in the set were already available from previous investigations and some others were designed and synthesized to have a panel of compounds with a modified oleanane carbon structure by adding a three-carbon side chain at C-3 with different lipophilicity, hydrogen bonding capacity, and flexibility.

## 2. Results and Discussion

### 2.1. Preparation of Oleanolic Acid Derivatives

**Scheme 1:**
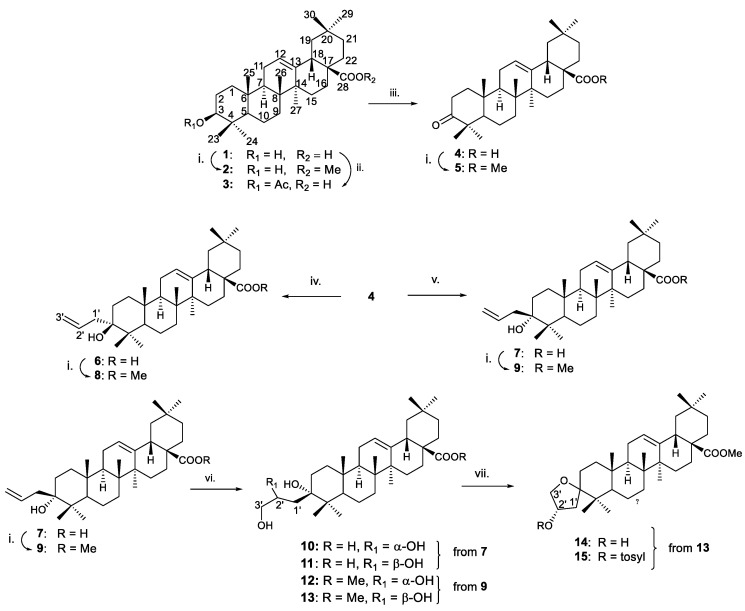
Reagents and conditions: i. CH_2_N_2_, diethyl ether, 0 °C, 30′; ii. Ac_2_O/Py 1:2 rt, overnight; iii. Jones reagent, acetone, 0 °C, 3 h; iv. allyl-MgBr, dry THF, 0 °C, 48 h; v. allyl-Br, In, THF, rt, 24 h; vi. OsO_4_ cat., NMO, acetone/H_2_O 6:1, rt, 48 h; vii. TsCl, TEA, DMAP, dry DCM, 48 h.

Methyl ester **2** was obtained by the treatment of compound **1** with an ethereal solution of CH_2_N_2_ at 0 °C. Acetate **3** was obtained from **1** with a standard acetylation procedure by treatment with Ac_2_O/Py, as reported previously [23]. The 3-oxoderivatives **4** and **5** were obtained by the oxidation of **1** [23] followed by CH_2_N_2_ methylation under the same conditions described above. The two epimeric 3-allyl derivatives **6** and **7** were prepared by Barbier–Grignard allylation with allyl magnesium bromide as previously reported [23]. Then, the new methyl esters **8** and **9** were obtained by the methylation of compounds **6** and **7**, respectively. The epimer **7** was obtained as a largely minor product in the Grignard reaction. Alternatively, **7** can be obtained, as we demonstrated within this work for the first time, by the Barbier–Grignard allylation of compound **4** performed with allyl bromide and indium in THF. This procedure is compatible with the presence of the ester function at C-28, which, on the other side, is incompatible with the strong basicity of the organo-magnesium reagents. Compounds **10** and **11** were prepared by osmylation, as described previously [1], while new compounds **12** and **13** were obtained by the same procedure applied on the methyl ester **9** (Scheme 1).

**Scheme 2:**
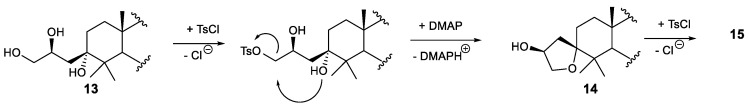
Formation of compounds **14** and **15** from **13**.

Finally, the two new spyro tetrahydrofuryl derivatives **14** and **15** were obtained by a new procedure developed in this work that implies the tosylation of the less hindered primary hydroxyl group in **13** to convert it into a leaving group suitable for intramolecular substitution accompanied by the spyrotetrahydrofuran-forming cyclization process depicted in Scheme 2.

To the best of our knowledge, this kind of cyclization is reported for the first time. Other methods are available to obtain this spyro system starting from an alkene that involves quite different conditions, i.e., the use of electrophilic reagents such as hypervalent iodine [24], dibutyltin oxide on triols [25], cerium chloride [26], or perchloric acid [27] on epoxyalcohols.

**Figure 1:**
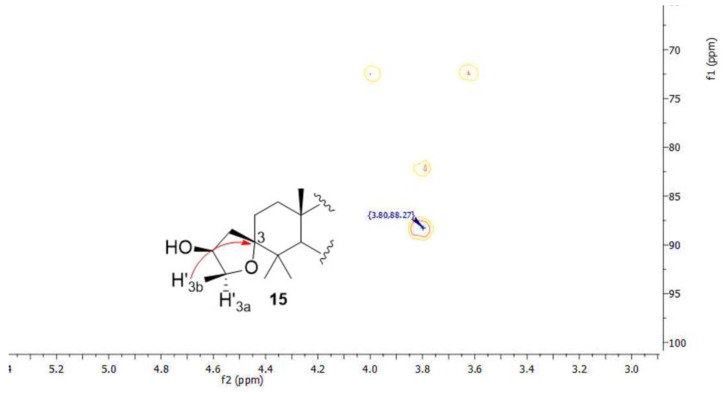
Long-range C-H hetero-correlation (HMBC) between the signals of C-3 (88.4 ppm) and H-3′_b_ on the side chain (3.81 ppm) of compound **15**.

All of the previously unknown compounds obtained in this work were fully characterized by complete spectroscopic analysis, particularly 1D and 2D NMR spectroscopy. The spectra of compounds **8**, **9**, **12**, and **13** were similar to those of their unmethylated counterparts and further exhibit the NMR resonances of the carbomethoxy fragment at *δ*_C_ 51.7, 51.7, 51.7, and 52.0 ppm, respectively, and *δ*_H_ 3.61, 3.62, 3.60, and 3.62 ppm in the above cited order (Table 1). The confirmation of the spyro-tetrahydrofuryl structure for compounds **14** and **15** was less trivial and was supported by the long-range C-H hetero-correlation, in particular between the signals of C-3 (88.4 ppm) and H-3′ on the side chain (3.81 ppm) (Figure 1).

### 2.2. Antibacterial Activity

All triterpene compounds were tested for their antibacterial activity against two different bacteria, with *E. coli* DH5α as a Gram-negative and *S. aureus* ATCC6538P as a Gram-positive model. 

**Figure 2:**
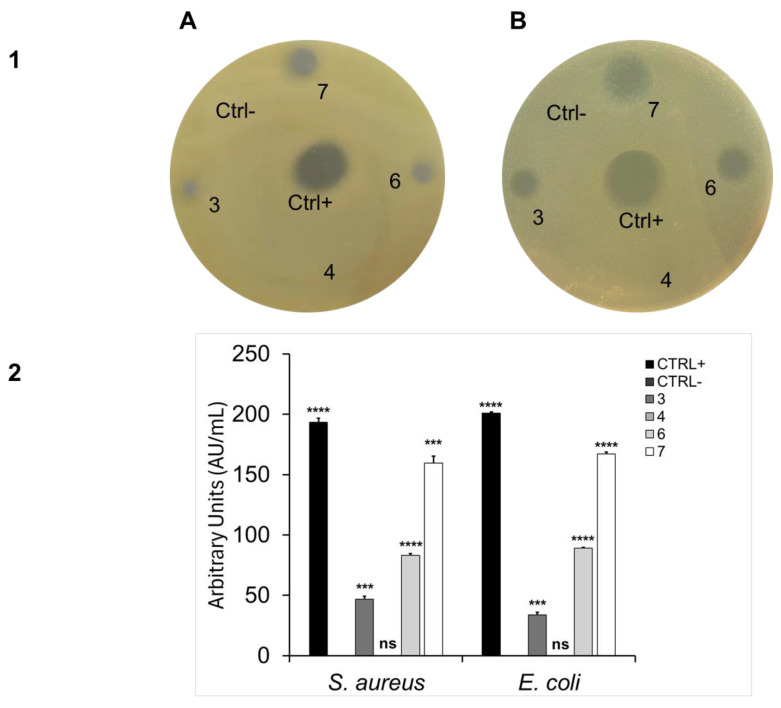
Bacterial growth inhibition: the inhibition halo of samples **3**, **4**, **6**, and **7** (panel 1) against (**A**) *S. aureus* and (**B**) *E. coli*. The positive control is ampicillin; the negative control is dimethyl sulfoxide. Panel 2 reports arbitrary units in milliliters for both bacterial strains. The assays were performed in three independent experiments (*n* = 3). Statistical analysis was performed using a two-tailed paired *t*-test (ns is not significant, *** *p* ≤ 0.005, **** *p* ≤ 0.001) versus the negative control.

The preliminary results, shown in Figure 2, highlight that compounds **6** and **7** were promising candidates for further investigation. 

As can be observed in Figure 2 (panel 1A against *S. aureus*, panel 1B against *E. coli*), these two compounds appear to have the highest antimicrobial activity, as can be seen from both the largest diameter halos (panel 1) and the graph of arbitrary units per milliliter (panel 2). It is known that the larger the diameter of the zone, the greater the antimicrobial potential of the tested molecule (as shown in Figure 2) [28]. Among the two terpenoids, it appears that **7** was the most active against both *S. aureus* and *E. coli*. It shows potential antibacterial activity almost comparable to the positive control, ampicillin. All other compounds exhibited poor antibacterial activity since, after the development of the assay, no significant inhibition zones were observed. Specifically, **3** and **4** behaved similarly to the negative control, DMSO, indicating a lack of antibacterial activity and above all indicating how the small structural variations on C-3 did not improve their antibacterial activity. Figure 2 does not show the effects of compounds **2**, **5**, **8–15**, as they did not result in inhibition. The literature confirms the biological importance of oleanolic acid (**1**). This triterpene is known for its antimicrobial, hepatoprotective, anti-inflammatory, antiallergic, antiviral, and cytotoxic activities. It is able to enhance the bioavailability of active ingredients of pharmaceutical formulations [29]. It has been found to be an active ingredient of *Olea ferruginea* Royle extract, to which antimicrobial activity is attributed. It has also been found to be inhibitory against the growth of intestinal bacteria such as *Bacteroides fragilis, Clostridium clostridiiforme, C. perfringens, C. paraputrificum, Escherichia coli, Enterobacter cloacae*, and *Salmonella typhimurium*. It shows antioxidant and pro-oxidant properties. It protects mammalian and bacterial cells from cytotoxicity induced by hydroperoxides [29].

To confirm the data obtained from the previous assay and to better investigate the intrinsic antibacterial activity of the various derivatives, the minimum inhibitory concentration (MIC) was estimated. The calculation of the MIC is essential for determining the efficacy of antibacterial agents against specific bacteria. In fact, it represents the lowest concentration of an antimicrobial, or in this case, a potential antimicrobial molecule, necessary to inhibit the visible growth of a specific bacterium. This parameter has various applications and significance in microbiology and medicine. For example, knowing the MIC can help personalize the dosage of antibiotics and/or antimicrobial molecules for patients, optimizing therapeutic efficacy and minimizing toxicity risks [30].

Compound **7** possessed a notable MIC value of 10 mg/mL. In contrast, samples **2**, **3**, **4**, **6**, **8**, and **9** exhibited a MIC higher than 10 mg/mL, as shown in Table 2. These results confirmed that the most promising sample is indeed sample **7**. This is worth noting as only the molecules bearing fewer polar and non-hydrogen-donating side chains are endowed of antibacterial activity. Further, the substitution of the hydrogen by the methyl group on the carboxy function (**8** vs. **6** and **9** vs. **7**) implies a complete loss of activity as shown by the complete lack of an inhibition zone for both **8** and **9.**

The lack of antibacterial activity of the natural precursor **1** toward *E. coli* confirms the results of Kuete et al. [31], while this is in contrast with the data reported by other authors that obtained a MIC of 50 µg/mL [32]. Similar differences in the reported evidence regarding the antibacterial activity of **1** toward *S. aureus* ATCC 25923 vary from the very good MIC value of 8 µg/mL [33] to 64 µg/mL [34]. Compound **1** was instead rather efficient against a number of other clinically relevant strains such as *M. tuberculosis* and *E. faecalis* [10].

It is also worth noting that the stereochemistry at C-3 is quite relevant for the antibacterial activity as it emerged by comparing the activities of **6** and **7**, the α-OH epimer being the most potent. For this reason, chemical modifications were devoted to obtaining further derivatives with 3-OH group with an α orientation. However, subsequent antibacterial tests at lower concentrations confirmed only compound **7** as the most promising lead compound, also confirming the presence of an apolar side chain in a *β* configuration as the best structural feature, together with the presence of the free carboxy functionality on C-28. 

### 2.3. Cytotoxic Activity

**Figure 3:**
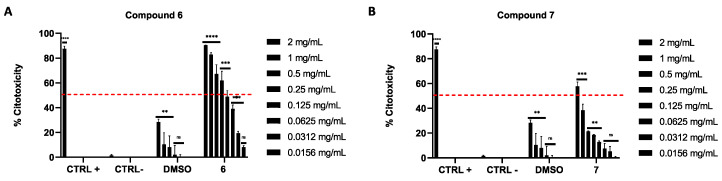
Cytotoxicity of **6** (**A**) and **7** (**B**) on the VERO-76 cell line after 24 h. The data represent the mean ± SD. Dunnett’s multiple comparisons test: **** *p* < 0.0001; *** *p* < 0.001; ** *p* < 0.01; ns *p* > 0.05.

The cytotoxic effects of **6** and **7** were performed on VERO-76 via the MTT assay. The cell monolayer was exposed to compounds ranging from 2 mg/mL to 0.0156 mg/mL for 24 h. DMSO (100%), used as a positive control (CTRL+), induced complete toxicity in the cell monolayer. On the other hand, cells treated with 2, 1, 0.5, 0.25, and 0.125% DMSO (corresponding to the solvent concentrations of DMSO in each treatment) showed cytotoxicity levels of about 28.3, 10.5, 8.1, 2.1, and 0.3%, respectively. Compound **6** recorded a cytotoxicity rate greater than 50% in the 2 to 0.25 mg/mL range. At 0.125 mg/mL the cytotoxicity was 48.9% and decreased in a dose-dependent manner at lower concentrations (Figure 3, histogram A). Compound **7** induced 57.8% cell death at 2 mg/mL, while at lower doses no relevant cytotoxic effect was verified (Figure 3, histogram B). Cytotoxicity data were normalized to the cell control rather than the solvent control, meaning that solvent cytotoxicity is included in the cytotoxicity data reported for each compound.

### 2.4. Antiviral Activity 

**Figure 4:**
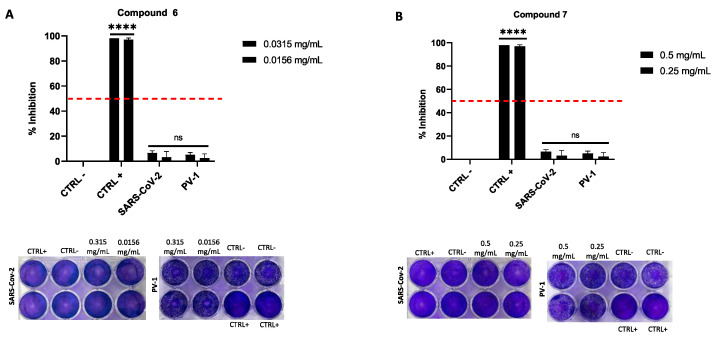
Antiviral activity of **6** (**A**) and **7** (**B**) against SARS-CoV-2 and PV-1. The data represent the mean ± SD. Dunnett’s multiple comparisons test: **** *p* < 0.0001; ns *p* > 0.05.

The antiviral efficacy of compounds **6** and **7** was evaluated in the co-treatment phase, through the plaque reduction assay in infected cells, against SARS-CoV2 and PV-1. Neither compound showed significant antiviral activity. In detail, at the concentrations tested (0.5 and 0.250 mg/mL for **7** and 0.031 and 0.015 mg/mL for **6**) the antiviral activity was less than 6% (Figure 4). Several studies have proven the antiviral activity of different terpenes against various viruses such as HIV, herpes simplex virus (HSV), hepatitis viruses, and influenza viruses. For example, betulinic acid, a well-studied triterpene, has shown a broad-spectrum antiviral activity, but its effectiveness can vary widely depending on modifications made to its structure and the specific viral strain [35]. For triterpenes that do not show antiviral activity, detailed studies and reviews often focus on their other pharmacological properties, such as cytotoxicity against tumor cells or anti-inflammatory effects [36,37]. These studies highlight that, although many triterpenes are potent antivirals, efficacy is not universal for all viruses or all triterpene derivatives.

## 3. Materials and Methods

### 3.1. General Procedures

Thin layer chromatography was performed on silica gel plates (Merck 60, F254, 0.2 mm) to monitor reaction progress. The NMR spectra were recorded on a Bruker Avance II 400 apparatus at 400.15 and 100.63 MHz for ^1^H and ^13^C, respectively, at the temperature of 300 K. The instrument was equipped with an inverse broadband probe (BBI). For the acquisition of ^1^H spectra, an 11.87 µs 90° pulse, a delay time of 5 s, and 16 scans were used. ^13^C spectra were acquired using a 90° pulse of 12.2 µs, a decoupling pulse of 80 µs, and a delay time of 3 s. The 2D NOESY spectra were acquired at the same temperature using a spin-lock pulse of 300 ms, eight scans, and 256 experiments. The 2D HSQC correlation spectra were acquired at the same temperature through a double inept transfer pulse sequence; the decoupling intervals were set as follows: a 90° pulse of 11.87 µs for ^1^H, with eight scans and 256 experiments. The stationary phase for flash chromatography (FC) was silica gel Merck (Kiesegel 60/230–400 mesh). Microanalysis data (C, H) were obtained by an Elemental Vario EI. III apparatus and are consistent with ± 0.4% of the theoretical value. Dry THF was obtained by suspension of THF onto 3 Å molecular sieves for 4 h. All chemical reagents were purchased from Merck s.r.l. (Sigma Aldrich, Milan, Italy). The solution of diazomethane in diethyl ether was obtained by Diazald® basic decomposition with KOH following the standard procedure [38,39].

### 3.2. Preparation of Oleanolic Derivatives

#### 3.2.1. Synthesis of Compounds **2**, **5**, **8**, and **9**

To a 0.5 M solution of the reactant compound in dry THF, an ethereal solution of CH_2_N_2_ was added dropwise at 0 °C until the resulting solution assumed a persistent pale-yellow color. Then, the volatiles were removed by the rotary evaporator and the residual was purified by chromatographic column giving compounds **2**, **5**, **8**, and **9** in quantitative yield (86.3, 94.7, 91.8, and 93.2%, respectively). The spectroscopic data of compounds **2** and **5** are in total agreement with the literature [40,41]. Methyl (3*α*)-allyl-(3*β*)-hydroxyolean-12-en-28-ate (**8**): white amorphous solid, anal. C 79.98%, H 10.70%; calcd. for C_34_H_54_O_3_; ^1^H NMR, and ^13^C NMR: see Table 1, Figures S1 and S2. 

#### 3.2.2. Synthesis of Compound **3**

An amount of 300 mg of compound **1** was dissolved in 6 mL of mixture of pyridine/acetic anhydride 2:1 and left to react overnight at room temperature (rt). Then, the volatiles were removed by co-evaporation with toluene and the residual was dissolved in 3 mL of THF and treated with 2 mL of NaHCO_3_ for 1 h in order to hydrolyze the residual mixed anhydride. The mixture was partitioned between water and CHCl_3_; the organic phase was dried onto Na_2_SO_4_ and the solvent was removed by rotary evaporation. The residue was purified by chromatographic column (CHCl_3_/acetone, 0.3% in acetone) giving 282 mg (86%) of pure **3**. The spectroscopic data of compound **3** is in total agreement with the literature [42].

#### 3.2.3. Synthesis of Compound **4**

To a 0.5 M solution of compound **1** in THF, an excess of Jones reagent was added dropwise at 0 °C until a light green persistent color developed. The insoluble residuals were removed by filtration and the solution was partitioned between water and CHCl_3_. The organic phase was neutralized with NaHCO_3sat_. and dried onto Na_2_SO_4_. The solvent was evaporated, and the residual was purified by chromatographic column (light petroleum/acetone 5% in acetone) giving pure **4** (80%). The spectroscopic data of compound **3** is in total agreement with the literature [43].

#### 3.2.4. Synthesis of Compounds **6** and **7**

(a) Allylmagnesium bromide procedure. To a 0.5 M solution of ketone **4** in dry THF, an excess of 2,5 eq. of a 2.0 M solution of allyl-MgCl in THF was added via syringe at 0 °C under an argon atmosphere. The resulting solution was stirred for 48 h and then the reaction was quenched by treatment with iced 1:1 HCl_aq_: until the pH of the mixture was adjusted to **2**. The mixture was extracted with CHCl_3_ and the resulting organic phase was neutralized with NaHCO_3sat_. and dried onto Na_2_SO_4._ The solvent was removed by the rotary evaporator and the residue was purified by chromatographic column (cyclohexane/acetone 9:1) giving pure **6** (45%) and **7** (10%). 

(b) Allyl bromide and indium procedure. An amount of 1 mmol of compound **4** was solubilized in 10 mL of THF and 600 µL (7.0 mmol) of allyl-Br and 800 mg (7.0 mmol) of powdered In were added in one portion. The resulting heterogeneous mixture was stirred vigorously for 24 h at 30 °C. Then, the reaction was quenched by adding 10 mL of 1:10 HCl_aq_. The mixture was extracted with CHCl_3_ and the organic phase was dried onto Na_2_SO_4._ The solvent was removed by evaporation and the residue was purified by chromatographic column (cyclohexane/acetone 9:1 to obtain pure **7**, 65%; cyclohexane/EtOAc 14:1 to obtain pure **6**). The spectroscopic data of compounds **6** and **7** are in total agreement with the literature [23,44].

#### 3.2.5. Synthesis of Compounds **10–13**

An amount of 0.5 mmol of the starting compounds **7** and **9** and 2.0 mmol (241.5 mg) of 97% *N*-methylmorpholine-*N*-oxide (NMO) were solubilized in 15 mL of acetone/THF/H_2_O 6:7:1 and treated with 1.5 mL (0.3 eq.) of a 2.5% *w*/*w* solution of OsO_4_ in in 2-methyl-2-propanol. The resulting solution was stirred for 48 h at rt and then quenched with 2 mL of Na_2_S_3_O_3sat._ The mixture was stirred for further 10 min and then extracted with CHCl_3_/EtOH 2:1. The organic phase was dried and evaporated giving a residue that was purified by chromatographic column (cyclohexane/EtOAc 1:1 to give pure **12**, 40% yield and **13**, 56% yield; cyclohexane/EtOAc 1:3 to give pure **10** and **11**). The spectroscopic data of compounds **10** and **11** are in total agreement with the literature [1].

Methyl (3*β*)-2′S,3-dihydroxypropyl-(3*α*)-hydroxyolean-12-en-28-oate (**12**): amorphous white solid, anal. C 74.98%, H 10.37%; calcd. for C_34_H_56_O_5_, C 74.96%, H 10.36%. ^1^H and ^13^C-NMR: see Table 1; Methyl (3*β*)-2′R,3-dihydroxypropyl-(3*α*)-hydroxyolean-12-en-28-oate (**13**): amorphous white solid, anal. C 74.98%, H 10.36%; calcd. for C_34_H_56_O_5_, C 74.96%, H 10.36%. ^1^H and ^13^C NMR: see Table 1, Figures S3–S8.

#### 3.2.6. Synthesis of Compounds **14** and **15**

The procedure to synthesized **14** as major compound: 300 mg (0.551 mmol) of compound **13** was dissolved in 30 mL of dry dichloromethane. Then, 1.05 gr (5.51 mmol, 10 eq.) of tosyl chloride, 766 µL (5.51 mmol, 10 eq.) of triethylamine, and 135 mg (1.1 mmol, 2 eq.) of 4-(*N*,*N*-dimethylamino)pyridine (DMAP) were added in one portion at rt. The mixture was stirred for 48 h, then a second portion of 10 eq. of DMAP was added and the mixture was stirred for further 6 h. Then, the reaction was quenched with 50 mL of H_2_O and extracted with CHCl_3_. The organic phase was dried, and the solvent was evaporated. The residue was purified by chromatographic column (cyclohexane/EtOAc 10:1) to give pure 14 (80%) and 15 (10%). 

In the procedure to synthesized **15** as a major component: 100 mg (0.184 mmol) of compound 13 was dissolved in 10 mL of dry dichloromethane. Then, 53 mg (1.5 eq.) of tosyl chloride, 25.5 µL (1 eq.) of triethylamine were added in one portion at rt. Then, the mixture was stirred for 72 h and then further 8.5 eq. of tosyl chloride, 9 eq. of triethylamine, and 2 eq. of DMAP were added. The reaction mixture was stirred for further 6 h and then quenched with 10 mL of water. The organic phase was dried, and the solvent was evaporated. The residue was purified by chromatographic column (cyclohexane/EtOAc 10:1) to give pure **14** (23%) and pure **15** (75%).

Compound **14**: amorphous white solid, anal. C 72.34%, H 8.90%; calcd. for C_41_H_60_SO_6_, C 72.31%, H 8.88% S 4.71%. ^1^H and ^13^C NMR: see Table 1; compound **15**: amorphous white solid, anal. C 77.55%, H 10.35%; calcd. for C_34_H_54_O_4_, C 77.52%, H 10.33%. ^1^H and ^13^C-NMR: see Table 1, Figures S9–S12.

### 3.3. Bacterial Strains and Antimicrobial Activity

To evaluate the antimicrobial activity on all compounds **1–15**, the Gram-negative *Escherichia coli* DH5α strain and the Gram-positive *Staphylococcus aureus* ATCC6538P strain were used. The antimicrobial properties of compounds **3**, **4**, **6**, and **7** were detected using the agar diffusion test following the Kirby–Bauer method [45] with some modifications. An amount of 5 μL of the various samples was placed on Luria–Bertani agar plates and then covered with ~10 mL of soft agar (0.7%) premixed with 10 μL of the *E. coli* and *S. aureus* strains. The negative control was dimethyl sulfoxide (DMSO 80%) used to resuspend the samples; the positive control was the antibiotic ampicillin (10 µL) at a concentration of 0.1 mg/mL. 

The plates were incubated overnight at 37 °C and the antimicrobial activity was evaluated by calculating arbitrary units based on the width of the halo formed, according to equation [46,47]:

$$AU/mL=\frac{Diameter\;of\;the\;zone\;of\;clearance\left(mm\right)\times 1000}{Volume\;taken\;in\;the\;well\;(\mu L)}$$

### 3.4. Determination of Minimal Inhibitory Concentration

The microdilution method established by the Clinical and Laboratory Standards Institute (CLSI) was used to determine the minimal inhibitory concentration (MIC) of the samples against the chosen strains. Briefly, 5 × 10^5^ CFU/mL of each bacterial strain were added to 95 µL of Mueller–Hinton broth (CAM-HB; Fisher Scientific, Segrate, Italy), supplemented or not with the various compounds (**3**, **4**, **6**, **7**, **8**, and **9**) used at various concentrations (0.5, 1, 2, 4, 8, or 10 mg/mL). DMSO was used as a negative control and the antibiotic ampicillin as a positive control [47]. After overnight incubation at 37 °C, MIC_100_ values were determined as the lowest concentration associated with no visible bacterial growth at 600 nm.

### 3.5. Cell Culture Condition

The cell line used for cytotoxicity testing originated from the renal epithelium of the African green monkey (*Cercopithecus aethiops*) (VERO-76 CRL-1587, Manassas, VA, USA). The cells were grown in Dulbecco’s modified Eagle medium (DMEM) containing 4.5 g/L glucose (Gibco Life Technologies, Paisley, UK), supplemented with 2 mM L-glutamine (Gibco Life Technologies, Paisley, UK), 100 IU/mL penicillin–streptomycin solution (Gibco Life Technologies, Paisley, United Kingdom), and 10% fetal bovine serum (FBS) (Gibco Life Technologies, Paisley, United Kingdom). For the assays, the cells were seeded into 96-well plates, with final volumes of 0.1 μL, respectively. Incubation occurred at 37 °C with 5% CO_2_ in a humidified environment.

### 3.6. Viruses

The viruses used in this study were poliovirus type 1 (PV-1 strain CHAT) and SARS-CoV-2, strain VR PV10734, kindly donated by the Lazzaro Spallanzani Hospital in Rome, Italy. Both viral strains were propagated on a VERO-76 cell line. The original titers (PFU/mL) were 10^8^ and 10^9^ plaque-forming units (PFU)/mL, for PV-1 and SARS-CoV-2, respectively.

### 3.7. Cell Cytotoxicity Assay

Cytotoxicity assessment was performed on all compounds (**1–15**) using the 3-[4,5-dimethylthiazol-2-yl]-2,5-diphenyl tetrazolium bromide (MTT) method. VERO-76 cells were meticulously seeded in 96-well plates at a density of 2 × 10^4^ cells/well in a final volume of 0.1 mL. Compounds **6** and **7** were subjected to serial dilutions, ensuring cell exposure to a concentration range of 2 to 0.156 mg/mL for 24 h. Cells cultured with DMSO and solvent represented positive (CTRL+) and negative (CTRL−) controls, respectively. After exposure, cell viability was assessed by adding 100 μL of MTT solution (0.03 mg/mL) to cell monolayers for 3 h at 37 °C. Then, the medium was removed, and the formazan salts were solubilized using 100 μL of DMSO. The cytotoxicity rate was quantified by measuring absorbance at 570 nm with a microplate reader (Tecan life science, Männedorf, SW).

### 3.8. Antiviral Activity Assays

The antiviral efficacy of triterpenes was assessed by co-treatment assays using plaque reduction assays in infected cells, as previously described [48,49]. VERO-76 cells were seeded at a density of 2 × 10^5^ cells/well in a 12-well plate and incubated overnight at 37 °C in a humidified environment with 5% CO_2_. The compounds were serially diluted in a concentration range of 500–250 mg/mL for **7** and 0.625–0.312 mg/mL for **6**. The cells were co-treated with viruses at a multiplicity of infection (MOI) of 0.01 and terpenes at selected concentrations for 1 h at 37 °C. Then, the virus–compound mixture was removed, and a complete medium (10% FBS) supplemented with 5% carboxymethylcellulose (Sigma, C5678, C5013) was added. The cells were incubated for 24 h at 37 °C and then fixed with 4% formaldehyde. Thus, the cell monolayer was stained with 0.5% crystal violet and the number of plaques was normalized to untreated infected cells. Pleconaril (2 µg/mL) and Ivermectin (10 μM) were used as positive controls (CTRL+) for PV-1 and SARS-CoV-2, respectively. 

### 3.9. Statistical Analysis

Antimicrobial assays were performed in biological triplicates and data were expressed as mean ± error standard (SE). The significance of the difference between treated samples and CTRL− for each assay was evaluated by two-tailed paired *t*-test. Cytotoxicity and antiviral assays were performed in biological and technical duplicates and data were expressed as mean ± standard deviation (SD). The significance of the difference between treated samples and CTRL− for each assay was evaluated by Dunnett’s test. For cytotoxicity and antiviral assays were used Graph Pad Prism 9.0 software (San Diego, CA, USA). A *p*-value < 0. 05 was considered significant.

## 4. Conclusions

In this study, we focused on the antibacterial and antiviral effects of different C-3 modified oleanolic acid derivatives. In particular, methylation, acetylation, oxidation, allylation, and osmylation reactions were performed; two new spyro tetrahydrofuryl derivatives were also prepared to evaluate the impact of these structural modifications on the biological activities investigated. Compound **7** is the most active antimicrobial, with a MIC value of 10 mg/mL. The stereochemistry of C-3 is quite relevant for the antibacterial activity, with the *α*-OH epimer **7** being the most potent compound. Also, the presence of a non-polar side chain in the *β* configuration, together with the presence of the free carboxyl functionality on C-28, is a fundamental structural feature necessary to preserve the antibacterial activity.

## Figures and Tables

**Table 1:** Spectroscopic data of compounds **8**, **9**, **12**, **13**, **14**, and **15** expressed in ppm (coupling constant J expressed in Hz).

Comp.	8		9		12		13		14		15	
C/H	C	H	C	H	C	H	C	H	C	H	C	H
1	36.9		36.7		36.9	1.25 m 1.27 m	34.4		34.8	1.29 m 1.31 m	34.7	
2	32.6		28.4		32.4	1.49 m 1.65 m	33.9		31.3	1.45 m 1.66 m	32.4	
3	75.0		76.2		77.1		75.9		85.1		88.2	
4	41.2		40.6		41.7		41.7		42.0		41.7	
5	50.9	1.21 m	51.1	1.19 m	51.5	1.17 m	51.0	1.18 m	51.6	1.24 m	51.5	1.22 m
6	18.8		18.9		18.8	1.37 m 1.43 m	20.8		18.7	1.40 m 1.42 m	18.8	
7	33.8		32.9		32.7	1.20 m 1.29 m	30.7		32.5	1.20 m 1.22 m	32.5	
8	39.2		39.3		39.2		37.0		37.1		36.8	
9	47.5		47.6		47.7	1.62 m	47.6		46.5	1.62 m	47.4	
10	30.7		37.1		36.7		36.8		31.2		30.9	
11	23.5		23.4		23.0	1.88 m 1.90 m	23.2		23.3	1.83 m 1.84 m	23.2	
12	122.4	5.29 t (3.4)	122.3	5.29 t (3.4)	122.3	5.29 t br (3.5)	122.2	5.28 t (3.5)	122.4	5.28 m	122.4	5.28 m
13	143.8		143.8		143.8		143.7		143.4		143.7	
14	41.7		41.6		40.8		39.2		40.7		40.4	
15	27.6		27.7		27.9	1.04 m 1.60 m	27.6		27.9	1.00 m 1.58 m	27.6	
16	23.2		23.1		23.5	1.61 m 1.96 m	23.0		23.2	1.60 m 1.87 m	23.0	
17	46.7		46.0		45.8		45.8		41.5		45.8	
18	40.9	2.86 dd (14.4, 4.4)	41.3	2.87 dd (14.0, 4.6)	41.2	2.85 dd (13.4, 4.3)	41.3	2.85 dd (13.9, 4.1)	41.3	2.85 m	41.2	2.84 dd (14.1, 4.3)
19	45.8		46.8		46.7	1.13 m 1.15 m	46.7		46.5	1.10 m 1.12 m	46.7	
20	30.7		30.7		30.7		30.2		30.8		30.7	
21	34.3		33.9		33.8	1.17 m 1.32 m	32.4		34.0	1.15 m 1.17 m	33.8	
22	32.4		32.4		34.4	1.20 m 1.45 m	32.7		29.2	1.65 m 1.73 m	29.1	
23	20.7	0.91 s	19.5	0.87 s	16.8	0.73 s	16.8	0.71 s	17.2	0.71 s	16.8	0.70 s
24	23.0	0.82 s	24.1	0.95 s	23.6	0.93 s	23.8	0.92 s	23.7	0.91 s	23.4	0.92 s
25	14.9	0.91 s	15.8	0.91 s	14.9	0.90 s	14.9	0.90 s	15.1	0.93 s	15.0	0.90 s
26	16.8	0.73 s	16.9	0.73 s	20.7	0.78 s	18.8	0.79 s	20.4	0.76 s	20.5	0.75 s
27	26.1	1.15 s	26.0	1.16 s	26.0	1.14 s	26.1	1.14 s	26.2	1.12 s	26.1	1.14 s
28	178.3		178.3		178.3		178.4		178.4		178.3	
29	33.1	0.93 s	33.1	0.91 s	33.1	0.90 s	33.1	0.90 s	33.2	0.91 s	33.1	0.89 s
30	23.6	0.94 s	23.6	0.93 s	23.7	0.94 s	23.6	0.92 s	23.8	0.76 s	23.6	0.92 s
1′	40.5	2.11 dd (13.8, 6.8) 2.41 dd (13.8, 8.2)	41.1	2.23 dd (13.5, 7.2) 2.48 dd (14.0, 7.4)	36.8	1.25 m 1.87 m	40.9	1.85 m 1.90 m	43.8	2.00 m 2.24 dd m (14.1, 6.6)	39.2	1.67 m 1.70 m
2′	134.7	5.93 ddt (17.1, 10.0, 7.3)	135.0	5.91 ddt. (17.0, 10.2, 7.5)	69.0	4.11 m	69.6	4.04 m	67.3	4.44 m	82.1	5.02 m
3′	118.9	5.12 m 5.16 m	118.0	5.10 dd (17.0, 2.3) 5.16 dd (10.2, 2.3)	67.4	3.44 dd (11.2, 6.7) 3.59 m	67.6	3.48 dd (10.9, 6.5) 3.58 m	78.2	3.81 m	72.3	3.81 m 3.90 m
1″											133.9	
2″ = 6″											129.9	7.34 d (8.5)
3″ = 5″											127.7	7.79 m
4″											144.8	
Tosyl-CH_3_											21.6	2.45 s
-OMe	51.5	3.63 s	51.5	3.62 s	50.7	3.63 s	51.6	3.62 s	51.5	3.62 s	51.5	3.62 s

**Table 2:** The minimal inhibitory concentration (MIC) of samples **3**, **4**, **6**, **7**, **8** and **9** against *E. coli*. The values were obtained from minimum of three independent experiments.

Strain	Compounds	MIC_100_ [mg/mL]
***E. coli* DH5*α***	**1**	>10
	**3**	>10
	**4**	>10
	**6**	>10
	**7**	10
	**8**	>10
	**9**	>10

All the other compounds were substantially inactive toward the bacterial strains tested.

## Data Availability

Data are contained within the article and Supplementary Materials.

## Supplementary Materials

The following supporting information can be downloaded at: https://www.mdpi.com/article/10.3390/ijms25158480/s1.
