# Supplementary material for: Biological Properties of Oleanolic Acid Derivatives Bearing Functionalized Side Chains at C-3
Source: Int J Mol Sci. 2024 Aug 3;25(15):8480. doi: 10.3390/ijms25158480 (PMC11312724; doi:10.3390/ijms25158480)
Supplement: Supplementary file 1 [file ijms-25-08480-s001.zip › ijms-3113822-supplementary.pdf]

Supplementary Materials

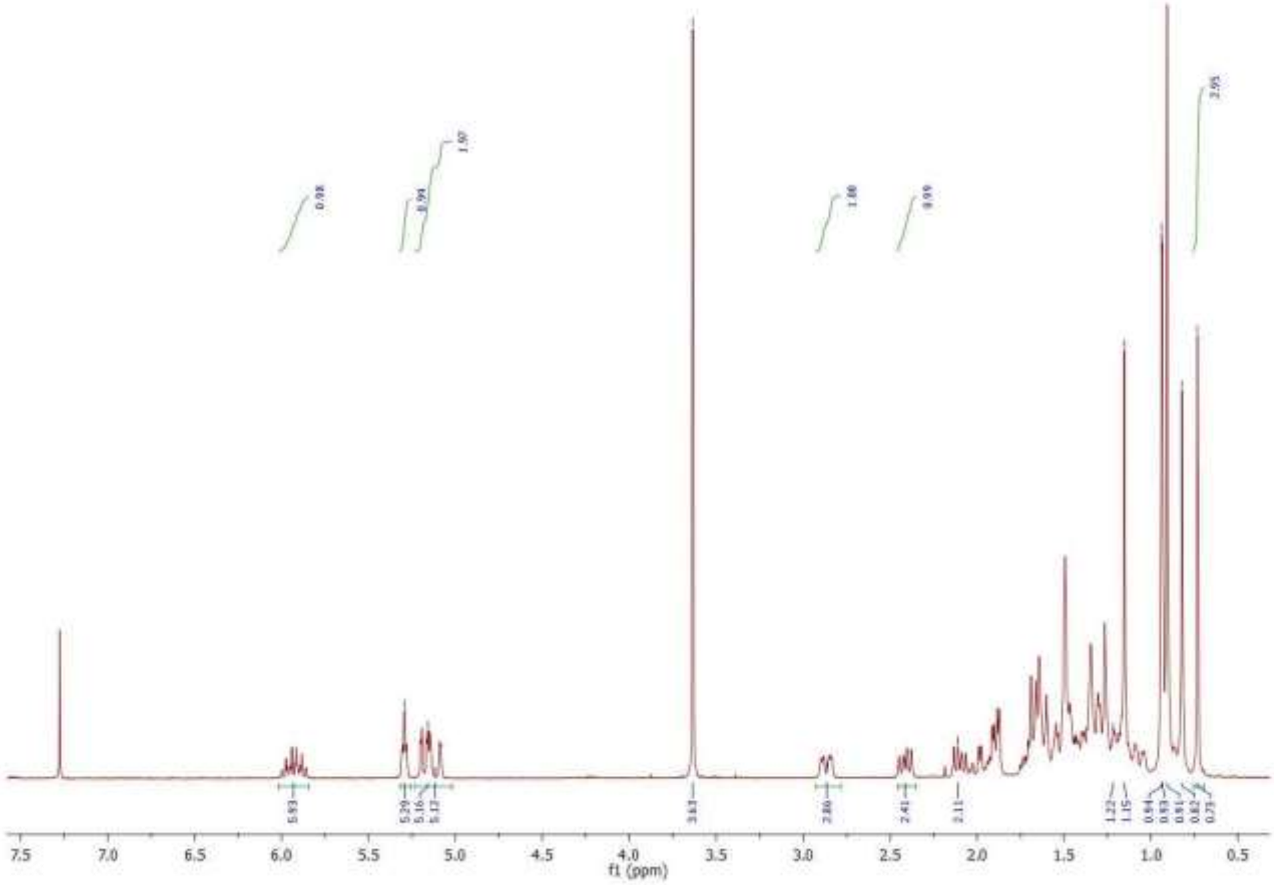

Figure S1. ^{1}H-NMR spectrum of compound **8**.

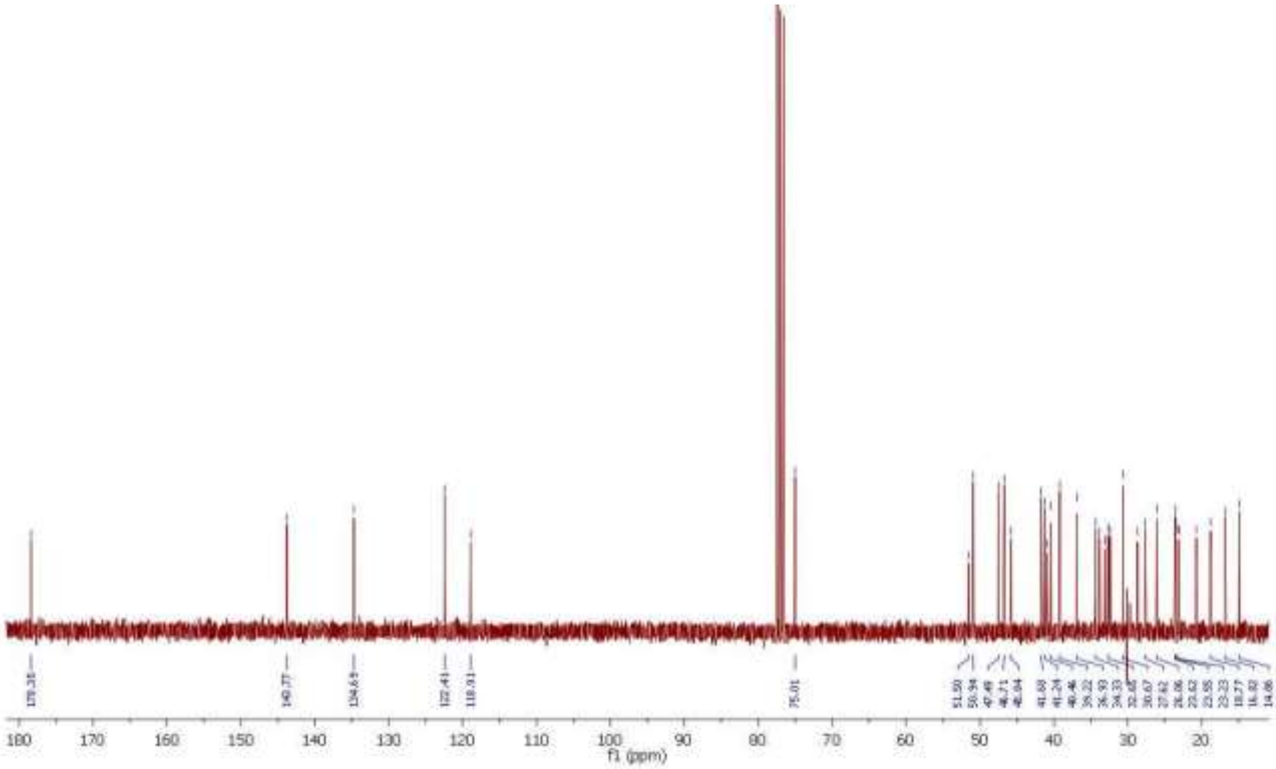

Figure S2. ^{13}C-NMR spectrum of compound **8**.

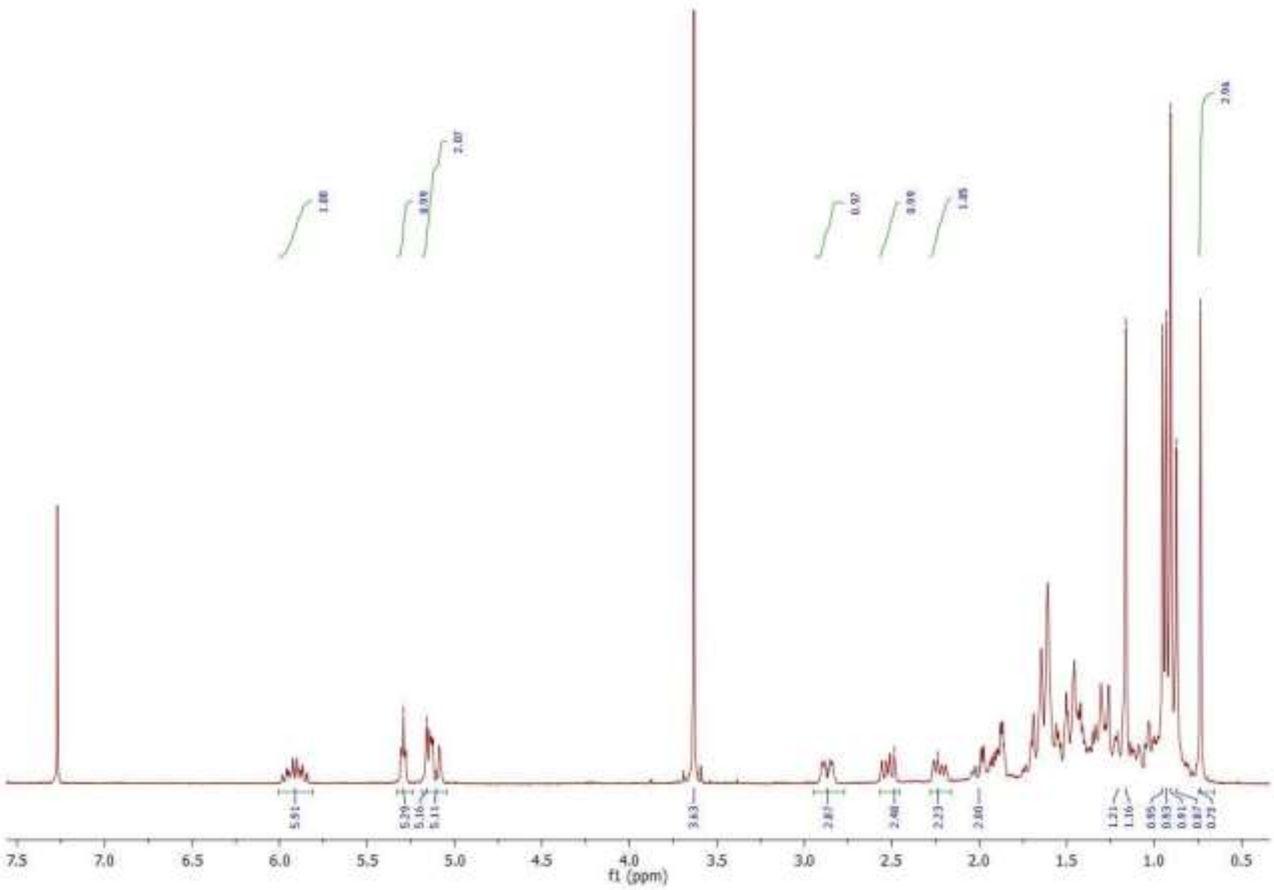

Figure S3. ^{1}H-NMR spectrum of compound **9**.

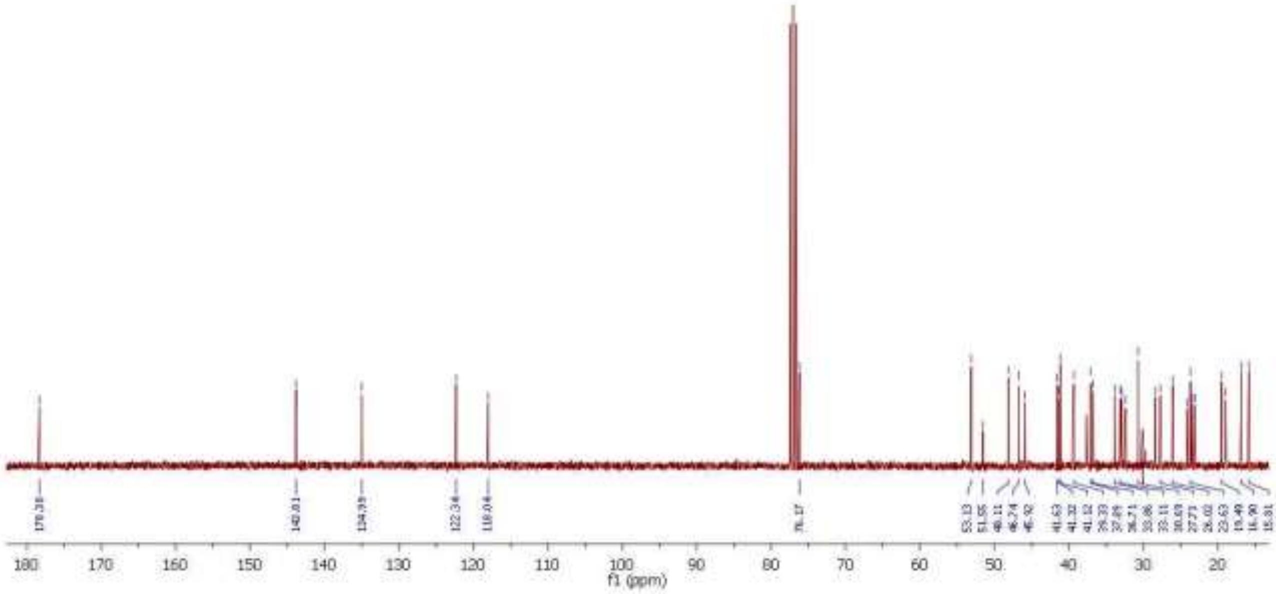

Figure S4. ^{13}C-NMR spectrum of compound **9**.

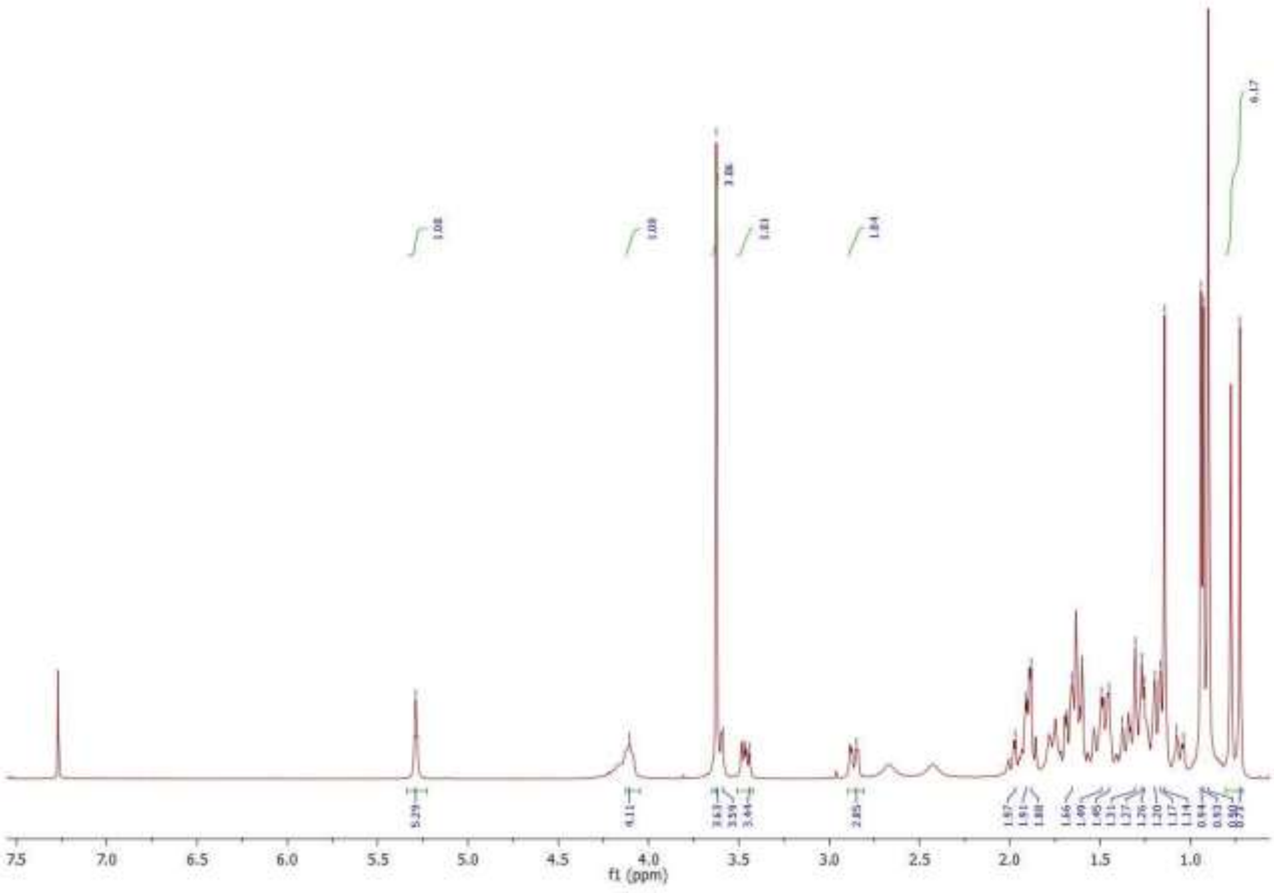

Figure S5. ^{1}H-NMR spectrum of compound **12**.

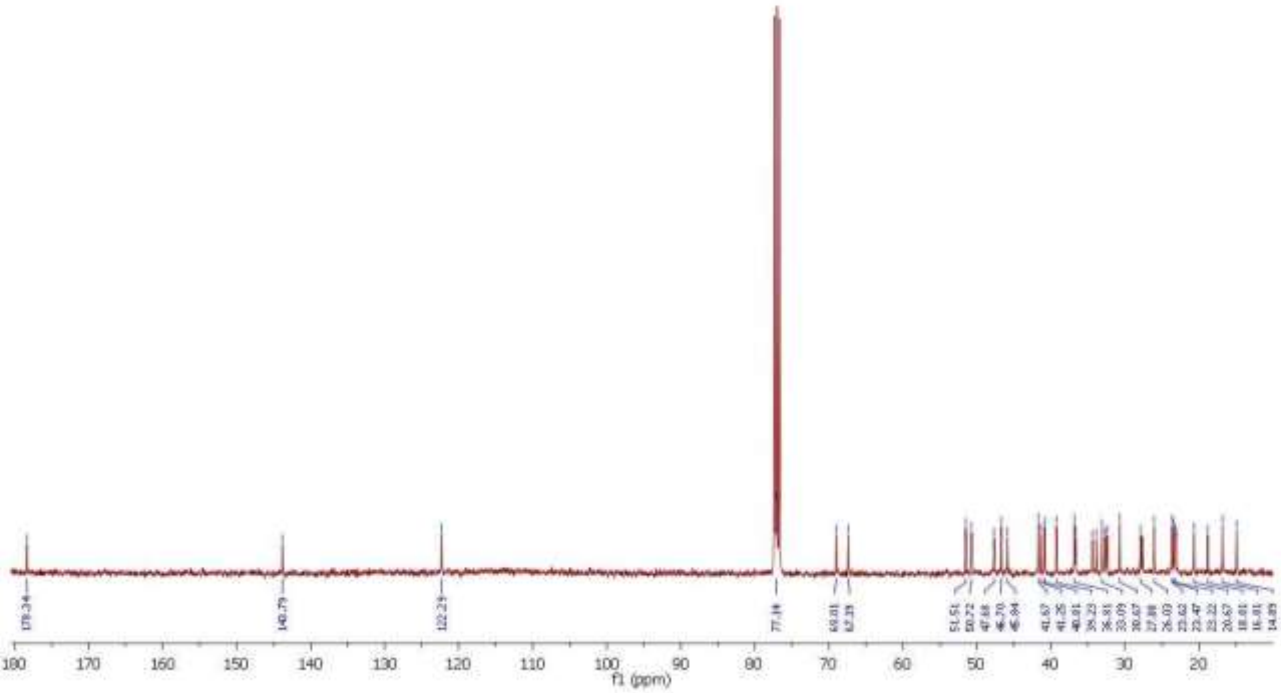

Figure S6. ^{13}C -NMR spectrum of compound **12**.

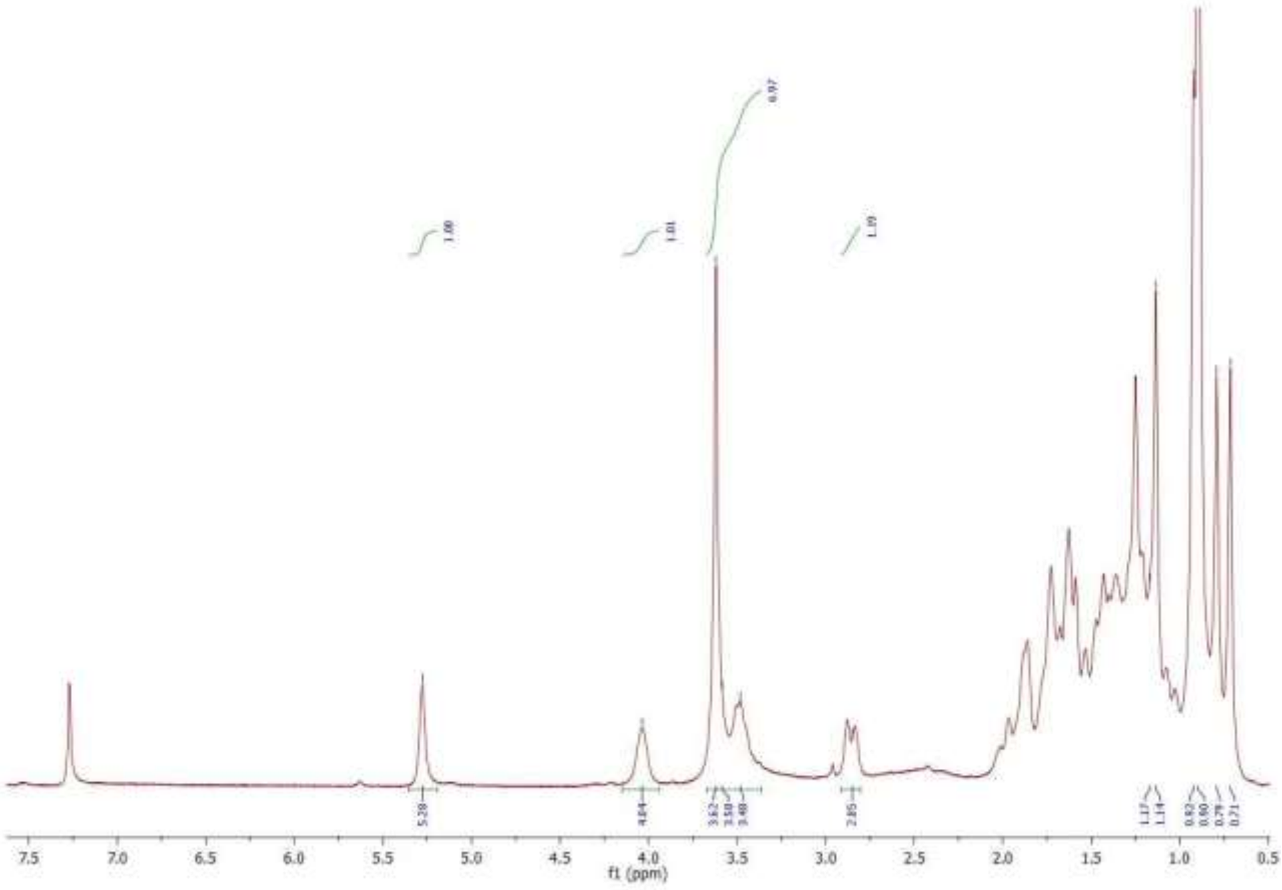

Figure S7. ^{1}H-NMR spectrum of compound **13**.

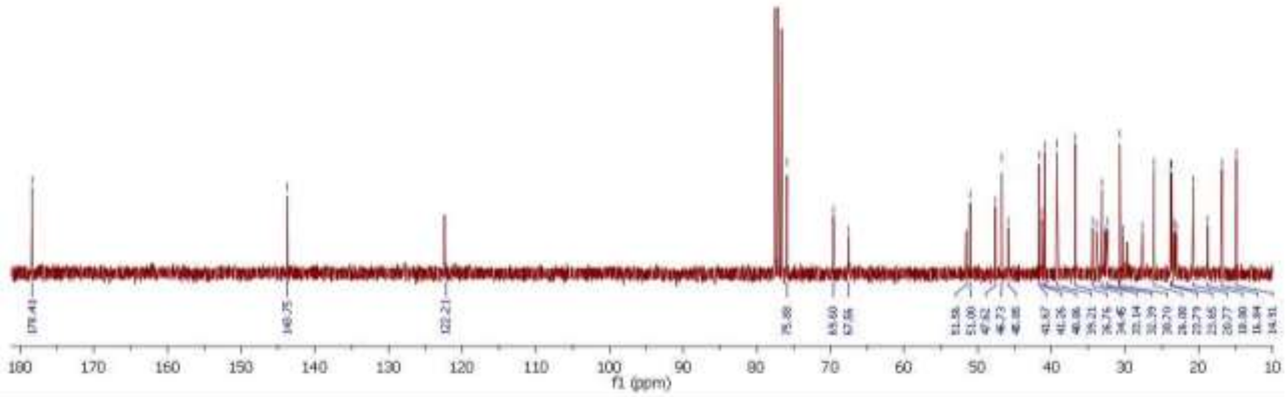

Figure S8. ^{13}C -NMR spectrum of compound **13**.

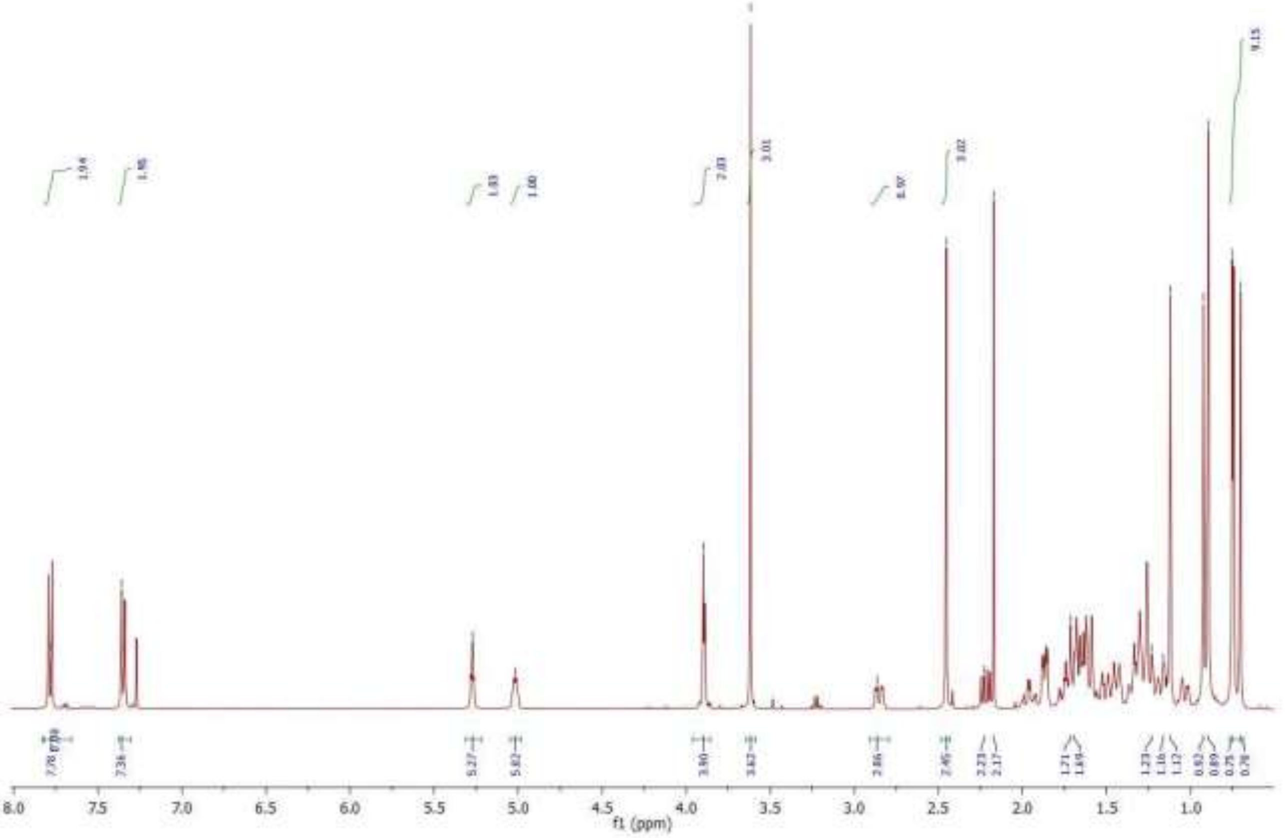

Figure S9. ^{1}H-NMR spectrum of compound **14**.

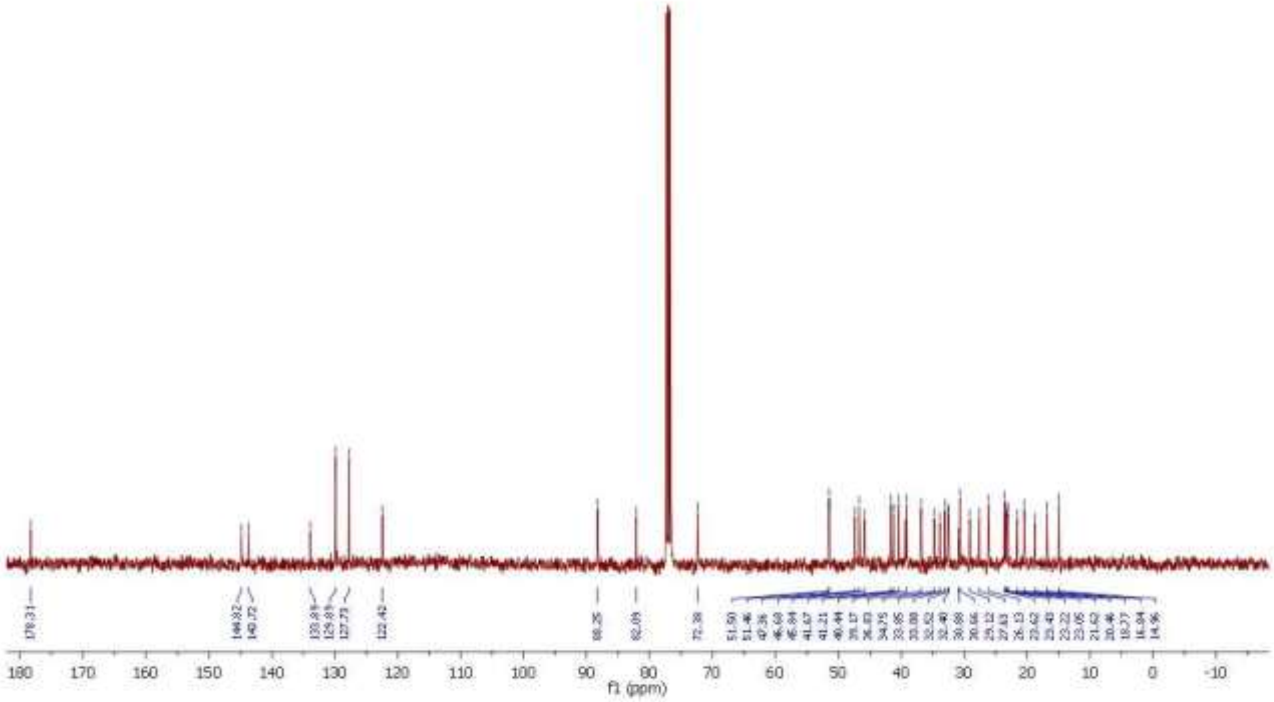

Figure S10. ^{13}C -NMR spectrum of compound **14**.

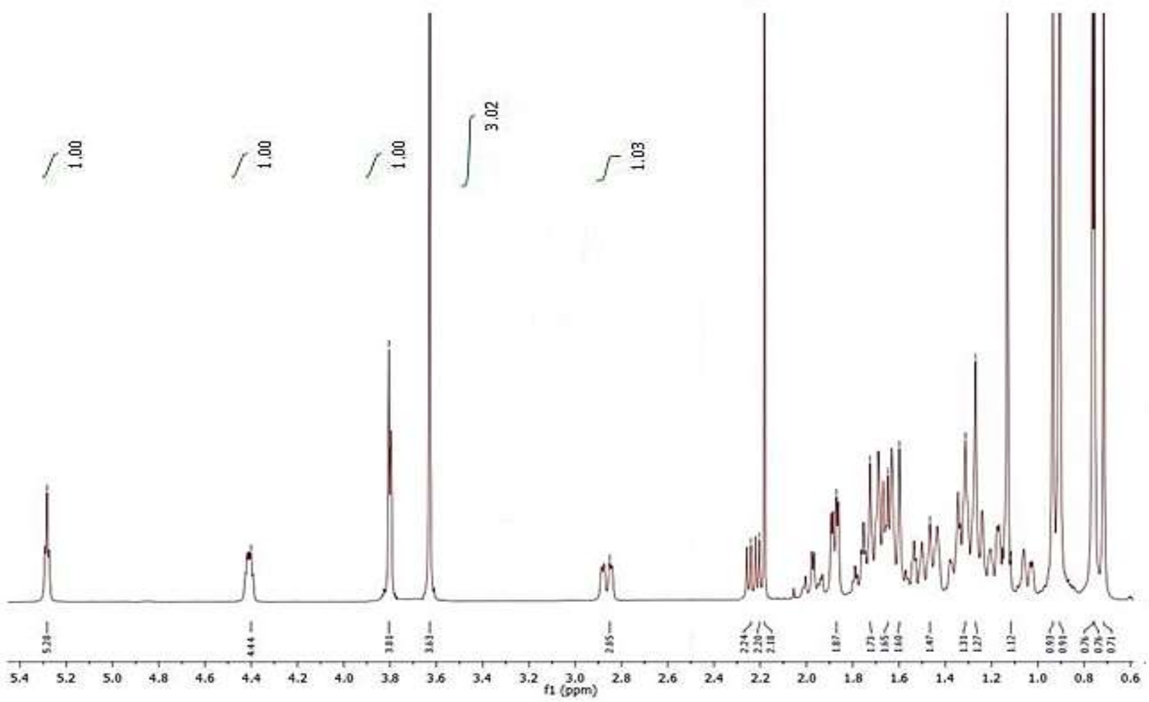

Figure S11. ^{1}H-NMR spectrum of compound **15**.

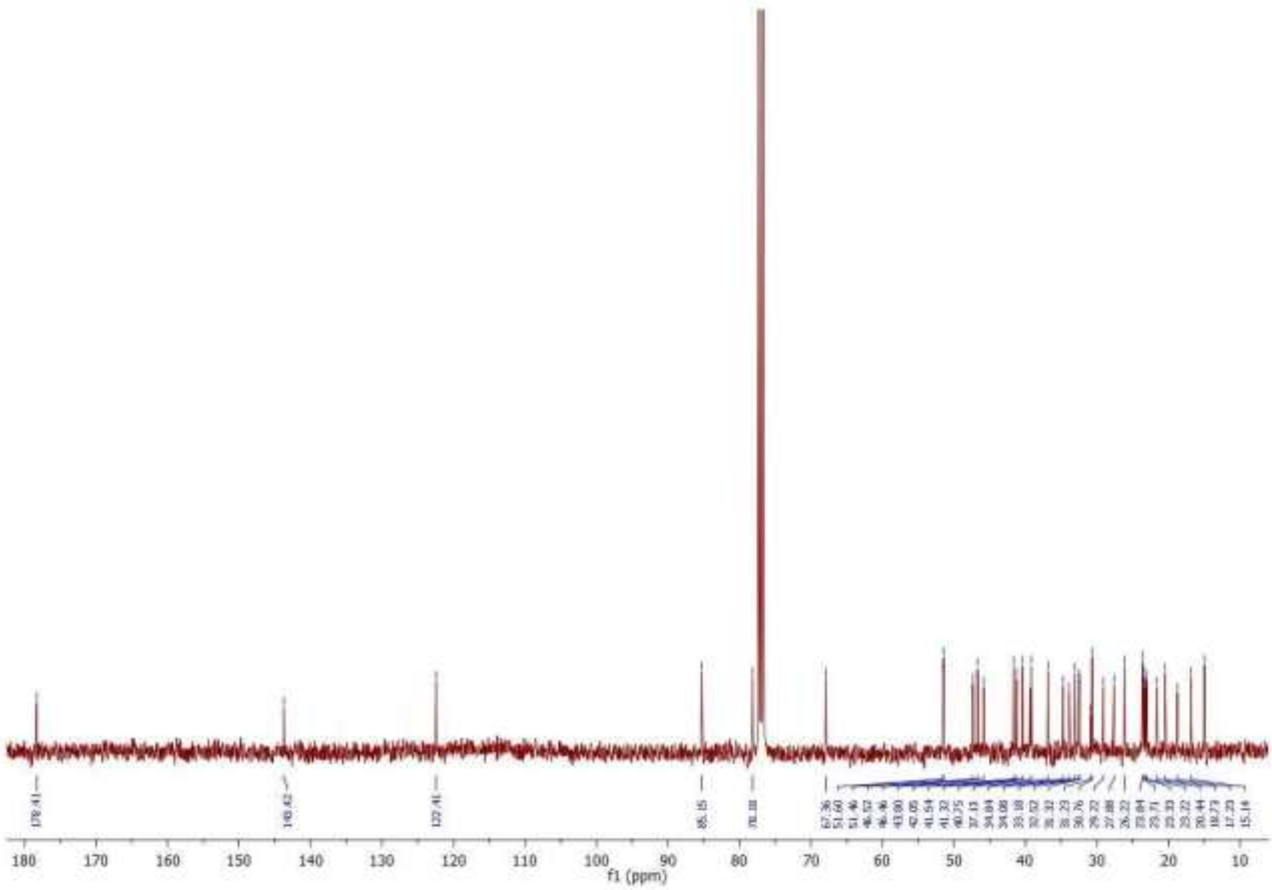

Figure S12. ^{13}C -NMR spectrum of compound **15**.
